# Comprehensive Ecological and Geographic Characterization of Eukaryotic and Prokaryotic Microbiomes in African *Anopheles*

**DOI:** 10.3389/fmicb.2021.635772

**Published:** 2021-05-12

**Authors:** Eugeni Belda Cuesta, Boubacar Coulibaly, Tullu Bukhari, Karin Eiglmeier, Raymond Kone, Mamadou B. Coulibaly, Soumanaba Zongo, Mamadou Barry, Awa Gneme, Wamdaogo M. Guelbeogo, Abdoul H. Beavogui, Sekou F. Traore, N’Fale Sagnon, Kenneth D. Vernick, Michelle M. Riehle

**Affiliations:** ^1^Unit of Insect Vector Genetics and Genomics, Department of Parasites and Insect Vectors, Institut Pasteur, Paris, France; ^2^CNRS Unit of Evolutionary Genomics, Modeling, and Health (UMR2000), Institut Pasteur, Paris, France; ^3^Malaria Research and Training Centre, Faculty of Medicine and Dentistry, University of Mali, Bamako, Mali; ^4^International Centre of Insect Physiology and Ecology, Department of Human Health. Nairobi,Kenya; ^5^Centre de Formation et de Recherche en Santé Rurale de Mafèrinyah, Conakry, Guinea; ^6^Centre National de Recherche et de Formation sur le Paludisme, Ouagadougou, Burkina Faso; ^7^Département de Biologie et Physiologie Animales, Université Joseph Ki-Zerbo, Ouagadougou, Burkina Faso; ^8^Department of Microbiology and Immunology, Medical College of Wisconsin, Milwaukee, WI, United States

**Keywords:** mosquito, *Anopheles*, insect microbiome, eukaryotic microbiology, commensalism, insect immunity

## Abstract

Exposure of mosquitoes to numerous eukaryotic and prokaryotic microbes in their associated microbiomes has probably helped drive the evolution of the innate immune system. To our knowledge, a metagenomic catalog of the eukaryotic microbiome has not been reported from any insect. Here we employ a novel approach to preferentially deplete host 18S ribosomal RNA gene amplicons to reveal the composition of the eukaryotic microbial communities of *Anopheles* larvae sampled in Kenya, Burkina Faso and Republic of Guinea (Conakry). We identified 453 eukaryotic operational taxonomic units (OTUs) associated with *Anopheles* larvae in nature, but an average of 45% of the 18S rRNA sequences clustered into OTUs that lacked a taxonomic assignment in the Silva database. Thus, the *Anopheles* microbiome contains a striking proportion of novel eukaryotic taxa. Using sequence similarity matching and *de novo* phylogenetic placement, the fraction of unassigned sequences was reduced to an average of 4%, and many unclassified OTUs were assigned as relatives of known taxa. A novel taxon of the genus *Ophryocystis* in the phylum Apicomplexa (which also includes *Plasmodium*) is widespread in *Anopheles* larvae from East and West Africa. Notably, *Ophryocystis* is present at fluctuating abundance among larval breeding sites, consistent with the expected pattern of an epidemic pathogen. Species richness of the eukaryotic microbiome was not significantly different across sites from East to West Africa, while species richness of the prokaryotic microbiome was significantly lower in West Africa. Laboratory colonies of *Anopheles coluzzii* harbor 26 eukaryotic OTUs, of which 38% (*n* = 10) are shared with wild populations, while 16 OTUs are unique to the laboratory colonies. Genetically distinct *An. coluzzii* colonies co-housed in the same facility maintain different prokaryotic microbiome profiles, suggesting a persistent host genetic influence on microbiome composition. These results provide a foundation to understand the role of the *Anopheles* eukaryotic microbiome in vector immunity and pathogen transmission. We hypothesize that prevalent apicomplexans such as *Ophryocystis* associated with *Anopheles* could induce interference or competition against *Plasmodium* within the vector. This and other members of the eukaryotic microbiome may offer candidates for new vector control tools.

## Highlights

- Microbes inhabit the animal digestive tract and body and are generally required for the health of the organism.
- *Anopheles* mosquitoes are responsible for significant human and animal mortality due to the pathogens they transmit.
- New vector control tools are needed because historically effective control methods are declining in effectiveness due to insecticide resistance and other factors.
- Characterization of the eukaryotic and prokaryotic microbes that inhabit mosquitoes could help identify new vector control tools, either as biological control agents or to interfere with pathogen infection and transmission by mosquitoes.

## Introduction

Mosquitoes carry a microbiome of associated eukaryotic and prokaryotic microbes, as well as the viruses that comprise the virome. This assemblage is thought to influence mosquito immunity and the transmission of mosquito borne pathogens, and some taxa could decrease mosquito longevity or pathogen transmission (Pumpuni et al., 1996; Ryu et al., 2008; Dong et al., 2009; Rodrigues et al., 2010; Cirimotich et al., 2011; Boissiere et al., 2012; Broderick et al., 2014; Carissimo et al., 2015; Nanfack-Minkeu et al., 2019; Mitri et al., 2020; Sharma et al., 2020). However, most characterization of the *Anopheles* microbiome to date has focused on the prokaryotic fraction, and the composition and biology of the natural eukaryotic microbiome remains essentially unknown.

Mosquitoes have a deep evolutionary history with the human and animal pathogens they transmit. Insects diverged from other arthropods more than a half billion years ago (Giribet and Edgecombe, 2012). At that time, the Apicomplexa, the phylum including the malaria parasite *Plasmodium*, were already old (Kopecna et al., 2006; Morrison, 2009). Apicomplexans were likely waterborne pathogens of arthropods, and are probably still found in mosquito larval breeding sites today. Thus, the foundational mechanisms of mosquito innate immunity, including the mechanisms addressed against *Plasmodium* today, probably evolved in mosquito common ancestors for protection from ancient arthropod pathogens (Medzhitov and Janeway, 1997; Hoffmann et al., 1999; Mitri et al., 2015; Nanfack Minkeu and Vernick, 2018). However, the candidate natural pathogens, particularly eukaryotic microbes similar to *Plasmodium* and other mosquito-transmitted pathogens, have not been systematically identified.

Profiling the prokaryotic microbiome is simple using amplicon sequencing of the 16S ribosomal RNA (16S rRNA) gene hypervariable regions. In contrast, profiling of the eukaryotic fraction of the microbiome is challenging because both the eukaryotic host as well as the eukaryotic microbiome carry highly related 18S ribosomal RNA (18S rRNA) genes, and the host contribution of 18S rRNA gene sequences in a DNA sample of the organism is in massive excess to that of the microbes. Here, we selectively enrich for amplification of 18S rRNA gene sequences originating in the eukaryotic microbiome. We used derivatized peptide-nucleic acid (PNA) oligonucleotides (called PNA blockers) that bind within the host 18S rRNA gene target and biochemically inhibit amplicon extension, thereby suppressing the generation of host-derived 18s rRNA gene sequences and enriching for the eukaryotic microbes (Belda et al., 2017).

Current vector control tools are being challenged by insecticide resistance and vector behavioral shifts. In the past decade, studies of the mosquito prokaryotic microbiome have led to potential new vector control approaches, including bacteria exhibiting *Plasmodium*-blocking phenotypes (Wang et al., 2017; Shane et al., 2018), the development of biopesticides from mosquito associated bacteria (Lacey, 2007; Caragata et al., 2020), or population replacement using *Wolbachia* (Ryan et al., 2019; Zheng et al., 2019). The eukaryotic members of the microbiome could also be useful to design similar or new approaches, such as interference or competition with *Plasmodium* superinfection, or as biological control agents. In order to explore these and other potential applications, a comprehensive assessment of the eukaryotic composition of the mosquito microbiome is first needed.

Here we sample wild mosquito larvae in West and East Africa, and also from laboratory colonies, in order to comprehensively characterize their eukaryotic and prokaryotic microbiomes using deep sequencing of 18S and 16S rRNA gene hypervariable region amplicons. We analyze association of microbiome parameters with mosquito species, geography, and larval breeding site ecology. Given the paucity of taxonomic database resources for eukaryotic microbes, we also implement an Evolutionary Placement Algorithm (EPA) for identification of many novel eukaryotic taxa.

## Materials and Methods

### Field Mosquito Samples

Larval samples were collected from the following sites. Samples were stored in 80% ethanol upon collection in the field and prior to DNA isolation.

#### Burkina Faso

Third and fourth instar mosquito larvae were collected near the village of Goundry in Burkina Faso during the rainy season in 2013. Different larval ecologies were sampled including mud brick pits, puddles, and ponds. Larval samples were stored in 80% ethanol prior to DNA isolation.

#### Republic of Guinea (Conakry)

Third and fourth instar mosquito larvae were collected across an ecological transect spanning dry savannah to deep forest ecologies in The Republic of Guinea and Mali during the rainy season in 2012 as previously described (Coulibaly et al., 2016). Larval samples were stored in 80% ethanol prior to DNA isolation.

#### Kenya

Third and fourth instar mosquito larvae were collected at three sites in the Luanda region of Kenya. Emutete village (34°64 E, 00°22 N) is in Emuhaya district in Western Kenya. It is a valley with slow running streams and considered lowland. Itumbu (34°57 E, 00°40 N) and Ebusilaro (34°60 E, 00°02 N) are also villages. However, they are closer to the town Luanda. In all three places, the households have farms that are cultivated almost year-round. The collections were done from a variety of breeding sites e.g., rain puddles, potholes on the roads, fish ponds, irrigation canals that were in the farms and dams for collecting rain water. Larval samples were stored in 80% ethanol prior to DNA isolation.

A summary table of all larval pools, their geographic locations and ecological attributes can be found in Supplementary Table 1.

### Laboratory Mosquito Samples

All laboratory samples were raised in the same insectary facility, exposed to the same water, food and other environmental variables, at the Institut Pasteur, Paris, France. The colonies M’bita, SDA500 and Ngousso are *An. gambiae, An. stephensi* and *An. coluzzii*, respectively, with origins in Kenya, Pakistan and Cameroon, respectively. The founder (Fd) and isofemale colonies (IML) including Fd03, Fd05, Fd09, Fd33 and IML26, IML29, IML30, IML30-2, and IML69 were previously described (Redmond et al., 2015) and originate from Burkina Faso and Mali. Briefly, Fd colonies were each initiated from the eggs of 6–11 wild-captured female mosquitoes that mated in nature. After oviposition, mothers were genotyped to determine species, and eggs of the same species were combined. IML colonies were initiated from a single mated female originating in an Fd colony.

### DNA Isolation

In addition to storage in 80% ethanol, all mosquito larvae were individually rinsed with 80% ethanol to remove surface microbes prior to DNA isolation. Genomic DNA was isolated from individual mosquitoes using DNAzol (Invitrogen, CA, United States). DNAs were resuspended in distilled water and stored at -20°C. All samples were typed by a molecular diagnostic assay to determine species status within the *An. gambiae* species complex (Fanello et al., 2002). In the event this assay failed to yield a diagnostic band, the ribosomal gene ITS2 region was PCR amplified, Sanger-sequenced, and the resulting sequence was used to search the NCBI nr database using blast. Mosquito species calls based on ITS2 sequence used a threshold of >98% nucleotide identity.

DNA pools comprised of 2–8 field-collected mosquito larvae each were constructed by pooling DNA from individual samples at equal volume. DNA pools were assembled after DNA isolation from individuals, because the species of each individual was first determined by molecular diagnostic assays prior to assembling DNA pools of the same species. From Burkina Faso, 17 DNA pools were each comprised of DNA from 2–7 larvae; from Republic of Guinea, 8 DNA pools were each comprised of DNA from 5–8 larvae; and from Kenya, 12 DNA pools were each comprised of DNA from 2–7 larvae. DNA pools were comprised of larval samples of the same mosquito species collected from the same geographic location and the same type of larval site. Water blank controls were co-processed, sequenced and analyzed with experimental samples. Resulting DNA pools were subjected to 18S and 16S rRNA gene amplicon sequencing as described below.

### Amplification of Hypervariable Regions of the 18S and 16S rRNA Genes

The prokaryotic and eukaryotic microbiomes of 39 DNA pools comprised of field collected samples and 24 DNA pools of laboratory colony mosquitoes (2 replicates for each of 12 colonies) were characterized by barcoding of the V4 hypervariable region of 16S and the V9 hypervariable region of 18S rRNA genes, respectively. Water blanks were also sequenced to detect any contamination. Samples were amplified using the following PCR recipe: 3 μl template DNA, 1.2 μl 5× KAPA HiFi buffer, 0.18 μl dNTP mix (10 mM), 0.3 μl DMSO, 0.003 μl 1,000× SYBR Green, 0.12 μl ROX (25 μM), 0.06 μl KAPA HiFi HotStart Polymerase (Kapa Biosystems), 0.3 μl V9 forward primer (10 μM), 0.3 μl V9 reverse primer (10 μM), 7.5 mM PNA blocker, nuclease-free water up to a reaction volume of 6 μl. The appropriate PNA blocker concentration was empirically determined previously (Belda et al., 2017). PNA blockers were incubated at 55°C for 5 min and vortexed to fully resuspend prior to adding to the reactions. Reactions were transferred into a 384-well plate and amplified with an ABI7900 thermocycler with the following amplification conditions: 95°C, 5 min and 25 cycles of: 98°C, 20 s, 78°C, 5 s, 55°C, 15 s, 72°C, 1 min. PCR products were diluted 1:100 in nuclease free water, and indexed using the procedure below.

### Library Construction From Amplified Products

Indexing PCR reactions were done using the following recipe: 5 μl template DNA, 1 μl nuclease-free water, 2 μl 5× KAPA HiFi buffer, 0.3 μl 10 mM dNTPs, 0.5 μl DMSO, 0.2 μl KAPA HiFi Polymerase, 0.5 μl forward primer (10 μM), and 0.5 μl reverse primer (10 μM). Indexing PCR reactions were carried out in 96-well plates on a Bio-Rad Tetrad two thermocycler, using the following cycling conditions: 95**°**C, 5 min and 10 cycles of: 98**°**C, 20 s, 55**°**C, 15 s, 72°C, 1 min, 72**°**C, 10 min. The following indexing primers were used (X indicates the positions of eight nucleotide unique indices for demultiplexing): Forward indexing primer: AATGATACGGCGACCACCGAGATCTACACXXX XXXXXTCGTCGGCAGCGTC. Reverse indexing primer: CAAGCAGAAGACGGCATACGAGATXXXXXXXXGTCT CGTGGGCTCGG.

### Library Normalization, Pooling, and Quantification

For the PNA blocker experiments, indexing PCR reactions were purified and normalized using a SequalPrep Normalization Plate Kit (Thermo Fisher Scientific). 10 μl of each sample was pooled (V4 and V9 were pooled separately, due to the different sizes of these amplicons) and the amplicon pools were purified and concentrated with a 1× AmPureXP (Beckman Coulter) clean up, followed by elution in 25 μl of Qiagen buffer EB.

The concentrations of the amplicon pools were determined using a Quant-iT PicoGreen dsDNA Assay Kit (Thermo Fisher Scientific) and amplicon sizes were verified on an Agilent Bioanalyzer High Sensitivity Chip. The V9 amplicon pools were independently diluted down to a 2 nM concentration in Qiagen EB buffer, and mixed at a 1:1 ratio.

### Library Denaturation, Dilution and Sequencing

10 μl of the 2 nM sequencing library was denatured by adding 10 μl of 0.2 N NaOH and incubating at room temperature for 5 min, then the library was diluted to 8 pM in Illumina HT1 buffer, spiked with 15% PhiX, and sequenced on a portion of a MiSeq 2 × 300 (600 cycle v3) lane. Library construction and sequencing was performed by the University of Minnesota Genomics Center, St. Paul, MN.

### Sequence Analyses

Raw paired-end reads were quality trimmed and assembled with Pandaseq (Masella et al., 2012). Primer regions were trimmed using the primer sequences rather than using a fixed value of Q. Following this, low quality amplicons were filtered out using quality scores defined as the geometric mean of their base qualities. Quality profile plots are shown in Supplementary Figure 1. Operational taxonomic unit (OTU) clustering and chimera filtering were carried out with QIIME version 1.9.1 (Caporaso et al., 2010b). Only 16S and 18S rRNA gene OTUs that contained more than 10 sequences were retained for subsequent analysis in order to avoid the inclusion of OTUs that were a product of sequencing error. Taxonomic assignment of 16S and 18S rRNA gene OTUs was carried out with QIIME version 1.9.1 using UCLUST (Edgar, 2010) against the 16S and 18S rRNA gene subdivision of the 119 release of the Silva database (Quast et al., 2013). The 16S rRNA gene OTU table was rarified to 10,000 reads per sample to correct for differences in sequencing depth with the *rarefy_even_depth* function of *phyloseq* R package, which was enough to observe saturation in rarefaction analyses (Supplementary Figure 2A). Nine field larval breeding sites were excluded after this step, which yielded DNA pools with very small fraction of amplicons joined after the Pandeseq step (mean 7.22% read pairs per DNA pool joined in full-length V4 16S rRNA amplicons in these 9 samples vs 88.99% in retained DNA pools). For 18S rRNA gene data, despite the use of PNA blockers, we observed a large number of sequences coming from the mosquito host, particularly in laboratory colony DNA pools (Supplementary Figure 3). After excluding OTUs coming from the mosquito host, the eukaryotic OTU table was rarified to 900 reads per sample with *rarefy_even_depth* function of *phyloseq* R package (McMurdie and Holmes, 2013), which was fixed based on rarefaction analyses (Supplementary Figure 2B). 7/24 laboratory colony DNA pools and 32/39 field DNA pools were retained for subsequent analyses (Supplementary Table 1). Diversity indexes (Observed species, Shannon, ACE, Chao1) were estimated from rarified OTU tables with the *estimate_richness* function of phyloseq R package (McMurdie and Holmes, 2013). The R package *vegan* (Oksanen et al., 2019) was used to compute Beta-diversity matrix from rarified OTU tables collapsed at genus level (*vegdist* function) and to visualize microbiome similarities using principle coordinate analysis (PCoA) (*cmdscale* function).

To identify covariates with the highest non-redundant explanatory power on 16S and 18S rRNA gene microbiota variation in mosquito DNA pools, first distance-based redundancy analyses was carried out on genus-level community ordination (PCoA based on Bray-Curtis beta-diversity matrix) with six pool covariates (country, mosquito species, 2La inversion, larval site description, larval breeding site status, larval site ecology) with *capscale* function of the *vegan* R package. Individual covariates significantly associated to variations in microbiome composition (*capscale p*-value < 0.05) were subsequently filtered to identify the ones with non-redundant explanatory power with the *env2fit* function of the vegan R package. Differential abundance analysis of prokaryotic and eukaryotic OTUs between conditions was carried out using the *phyloseq* implementation of DESeq2 method (Love et al., 2014). Water blank samples for 18S rRNA gene amplicon sequences included six OTUs, none of which were present among the OTUs from the experimental samples analyzed in the manuscript, and the 16S rRNA gene amplicon sequences included ten OTUs (Supplementary Figure 4).

### Phylogenetic Analysis

In order to improve the taxonomic annotation of eukaryotic OTUs, 38 unclassified OTUs with at least 100 sequence reads each were placed in a reference phylogeny of 18S rRNA sequences using the EPA of RAxML, which sequentially places each short query sequence (read) at each edge of a reference tree previously constructed with longer sequences and calculates the likelihood of the resulting tree (Berger et al., 2011). For this purpose, the OTUs were aligned with Pynast (Caporaso et al., 2010a) against a curated 18S rRNA sequence alignment template from release 119 of the Silva database (Quast et al., 2013), and the resulting alignment was filtered with the *filter_alignment.py* script of QIIME version 1.9.1 (Caporaso et al., 2010b). This filtered alignment was used to place unclassified eukaryotic OTUs in the reference 18S rRNA tree from release 119 of the Silva database (Quast et al., 2013), using the EPA of RAxML (Berger et al., 2011).

## Results

Here we characterize the eukaryotic and prokaryotic microbiomes of *Anopheles* larvae sampled in three countries of East and West Africa: Kenya, Burkina Faso and the Republic of Guinea. For detection of eukaryotic taxa, we implemented a PNA blocking strategy combined with deep sequencing of 18S rRNA gene amplicons to suppress mosquito sequence reads, a technique we have optimized previously (Belda et al., 2017). We also profile the prokaryotic microbiome in the same samples because of the technical simplicity of 16S rRNA gene amplicon sequencing, but most analysis herein is focused on the eukaryotic microbiome, which is novel.

### Composition of Eukaryotic and Prokaryotic Microbiomes

**FIGURE 1:**
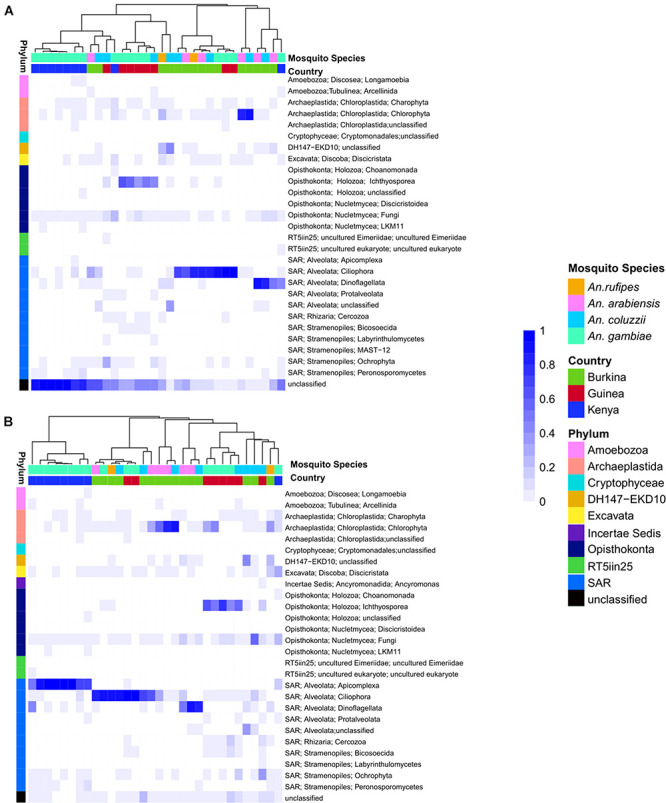
**(A)** Heatmap depicting the similarity in composition of micro eukaryotic members of the microbiome from field collected larvae displays clustering due to geography. The heatmap is clustered by microbiome similarity and both mosquito species and country of origin are shown above the heatmap for each sample with keys to the right of the heatmap. The dendrogram above the heatmap depicts overall micro eukaryotic microbiome similarity. The colored vertical bar on the left of the heatmap depicts the OTU Phylum membership with a key to the right of the heatmap. Each column represents a single pool of mosquito larvae subjected to 18S rRNA gene V9 amplicon sequencing and each row of the heat map show the relative proportion of one eukaryotic Order. The shade of blue indicates the proportion of the microbiome occupied by a particular Order; key to right of heatmap. **(B)** As in A except after application of the Evolutionary Placement Algorithm (EPA) which was used to assign many of the previously unassigned sequence reads to known phyla based on evolutionary relatedness as described in greater details in the methods. This phylogenetically informed process assigned on average 34.8% of previously unclassified sequences per field sample to known Orders.

The eukaryotic microbiome displays clear sample clustering by country. Specifically, many samples from Burkina Faso cluster together, and all but one Kenyan sample cluster with other samples from Kenya (Figure 1A). The most striking observation regarding the eukaryotic fraction of the microbiome is the large number of unclassified operational taxonomic units (OTUs) that do not match any entry in the Silva taxonomic database (the row marked “unclassified” at the bottom of Figure 1A). The deepest split in the sample dendrogram separates mosquito larval samples with a large fraction of unclassified OTUs from those that harbor a large fraction of Alveolata. The Alveolata are a major clade of protists that include the phylum Apicomplexa, to which *Plasmodium* belongs.

Among the classified taxa associated with mosquito larvae, some are notable for their abundance in particular samples. Larvae from Burkina Faso harbored high numbers of members of Ciliophora, a phylum of ciliated protozoans within the Alveolata that includes commensal as well as parasitic species, and Chlorophyta, a phylum of green algae that might be present as a larval food source, although there are also commensal and pathogenic species. Larvae from Guinea also displayed high abundance of Ciliophora, as well as Ichthyosporea, a group of Opisthokonta that are mostly parasites, discussed further below. In contrast, the larval samples from Kenya harbored a large proportion of OTUs lacking taxonomic assignment in the Silva database (Supplementary Table 2).

In order to extract additional information from the unclassified eukaryotic OTUs, we applied an EPA to classify unidentified OTU sequences based on evolutionary similarity to known OTUs. Prior to analysis using the EPA, an average of 39% (range: 1–98%) of eukaryotic sequence reads from a given sample lacked taxonomic assignment. In contrast, following analysis using the EPA the percent of unassigned reads dropped to 4% (range: 0–25%). The EPA analysis identified an average of 23% of previously unclassified eukaryotic OTUs as related to known OTUs across both field and laboratory samples (Figure 1B). Of the 27 field samples harboring unclassified eukaryotic OTUs at >5% abundance, analysis using the EPA improved taxonomic assignments in 19 samples, leaving only eight field samples with >5% unclassified reads at the OTU level.

Among the previously unclassified eukaryotic OTUs placed using EPA analysis, many are novel members of the order Alveolata, followed by a large proportion of novel members of the Chloroplastida (Figure 1B). In particular, almost all of the unclassified eukaryotic OTUs in the Kenyan field samples were taxonomically assigned by EPA analysis to the phylum Apicomplexa. The apicomplexan sequences are clustered as OTU38 and placed by EPA analysis as a novel taxon in the genus *Ophryocystis*, hereafter referred to as OTU38_Ophryocystis (Supplementary Table 3). Species of *Ophryocystis* have been described as insect pathogens in at least butterflies and beetles (Yaman and Radek, 2017; Gao K. et al., 2020). Application of the EPA also assigned additional OTUs in the Guinea samples to *Ochrophyta*, a group of photosynthetic heterokonts, and also assigned additional OTUs in the Burkina Faso and Guinea samples to the Peronosporomycetes clade in the phylum Oomycota, a group of fungus-like parasites and saprophytes known as water molds.

**FIGURE 2:**
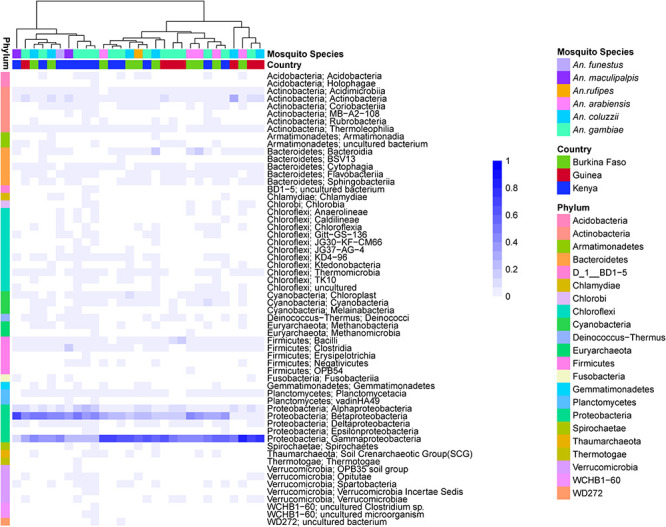
Heatmap depicting the similarity in prokaryotic microbiome composition of field collected larvae shows Betaproteobacteria and Gammaproteobacteria are common members of the prokaryotic microbiome across sampling countries and mosquito species. The heat map is clustered by microbiome similarity and both mosquito species and country of origin are shown above the heatmap for each sample. The dendrogram above the heatmap depicts overall prokaryotic microbiome similarity. The colored vertical bar to the left of the heat map highlight the Phylum membership for identified OTUs with a key to the right of the heatmap. Each column represents a single pool of mosquito larvae subjected to 16S rRNA gene V4 hypervariable region amplicon sequencing. Each row of the heat map indicates the relative proportion of one prokaryotic Class. The shade of blue indicates the proportion of the microbiome occupied by that particular Class level with a key to the right of the heatmap.

For the prokaryotic fraction of the microbiome, two families of gram-negative *Proteobacteria*, the *Betaproteobacteria* and *Gammaproteobacteria*, are prevalent across all field captured samples. The deepest root in the dendrogram depicting sample similarity is explained by the distribution of these two families. Many other bacterial phyla are present in all microbiome samples including gram-positive *Firmicutes* and *Actinobacteria*, while individual bacterial families within the gram-negative Chloroflexi phylum tend to be present in just a few samples (Figure 2). There is little apparent clustering of the prokaryotic microbiome by either country of origin or mosquito species (Supplementary Table 4).

### Structuring Influences on Wild *Anopheles* Microbiome Composition

**FIGURE 3:**
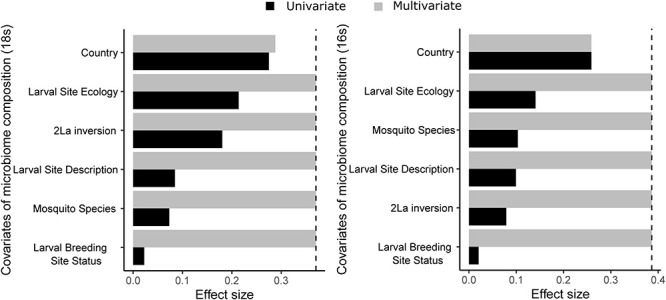
Country of origin is the sample variable showing the greatest correlation with microbiome composition (dbRDA, genus-level Bray-Curtis dissimilarity) for both 18S rRNA gene data (left) and 16S rRNA gene data (right) from field collected samples. Data is shown for both an independent model (black bars; univariate effect sizes, CAP_r2ad) and a multivariate model (gray bars; cumulative effect sizes, RDAcumul_R2.ad). The cut-off for significant non-redundant contribution to the multivariate model is shown by the vertical dashed line. Larval site ecology is the ecology of the geographic region (i.e., deep forest, dry savannah etc.), larval site types are puddle, pond, mud brick pit etc. and larval pool status is whether the pool is temporary, semi-permanent or permanent.

The major correlates of *Anopheles* microbiome compositional differences were determined by testing six attributes of the collected mosquito samples (Figure 3). Comparison of genus-level beta-diversity of microbial taxa using the Bray-Curtis dissimilarity statistic indicates that the country of sample collection, a proxy for the most coarse-grained geographic definition of the samples, displays the greatest correlation with the composition of both the eukaryotic and prokaryotic microbiomes. After the variable, country, the second most correlated attribute is larval site ecology (deep forest, dry savannah, etc.), a definition that is shared among and is independent of country of origin. These two attributes, country and larval site ecology, make non-redundant contributions to the multivariate model for either eukaryotic or prokaryotic microbiome. Interestingly, mosquito species displayed little correlation with microbiome composition. Thus, relatively little influence on the microbiome is seen from genetic differences between *Anopheles* species, or 2La chromosome inversion genotypes for *An. gambiae* and *An. coluzzii*, nor from larval breeding site substrate type or permanence. These results indicate that the most important structuring influences on the taxonomic composition of both eukaryotic and prokaryotic microbiomes harbored by *Anopheles* larvae are high-order ecological factors, defined by country of origin and, independently of country, the ecological characteristics of larval sites.

**FIGURE 4:**
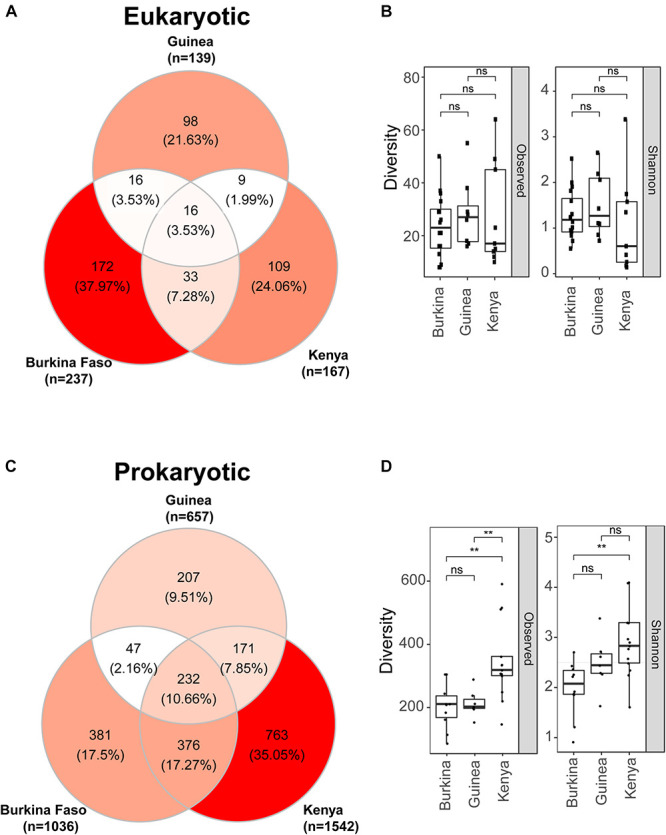
Eukaryotic microbes detected in mosquito larvae are more unique to the geographic location of collection than prokaryotic microbiomes where individuals OTUs are more often detected in mosquito larvae from at least two locations. **(A)** Venn diagram depicting the number of eukaryotic OTUs detected in DNA pools of mosquito larvae across Burkina Faso, Guinea and Kenya. **(B)** Eukaryotic microbiome diversity as a function of geographic origin shown as both observed OTUs (left) and Shannon diversity (right). There is no significant difference in eukaryotic diversity as a function of country of origin; ns = non-significant, *p* > 0.05. For these box plots the upper and lower bounds of the box indicate the third and first quartiles, respectively, and the line within the box indicates the median. Whiskers extends to the largest values no further than 1.5 time the inter-quartile range from the corresponding upper and lower bounds of the box. All individual data points are shown as individual dots. **(C)** Venn diagram depicting the number of prokaryotic OTUs detected in DNA pools of mosquito larvae and shared across Burkina Faso, Guinea and Kenya. **(D)** Prokaryotic microbiome diversity as a function of geographic origin shown both as observed OTUs (left) and Shannon diversity (right). Shannon diversity takes into account both richness and evenness while Observed diversity considered only richness. Mosquito larvae sampled in Kenya have significantly greater prokaryotic diversity measured by either metric, **, *p* ≤ 0.01. Box plots as in B.

We next compared on a finer scale the eukaryotic and prokaryotic microbiomes across the three sampled countries. Composition of the eukaryotic microbiome displays little similarity across the countries sampled, with only 4% of OTUs present in larval samples from all three countries (Figure 4A). An additional ∼12% of OTUs were present in *Anopheles* larval samples sampled from any two of the three countries. Overall, most of the eukaryotic OTUs (84%) were detected in only one country. *Anopheles* larvae from Burkina Faso display the greatest number of unique eukaryotic OTUs. Eukaryotic microbiome diversity does not significantly differ across countries, but samples from Kenya show a greater range in Observed and Shannon diversity values (Figure 4B) (Supplementary Table 5).

For the prokaryotic microbiome, *Anopheles* samples from Kenya displayed the greatest taxonomic diversity as well as number of unique OTUs (Figure 4C). Approximately 10% of prokaryotic OTUs were shared across all three countries, and an additional 27% of OTUs were shared across any two countries. Thus, the majority of OTUs identified (63%) were sampled from only one country (Figure 4D), a finding similar to the eukaryotic microbiome. Diversity of the prokaryotic microbiome measured by either observed or Shannon diversity indicate that Kenyan *Anopheles* larvae display significantly greater prokaryotic microbiome diversity than those from Burkina Faso, while prokaryotic diversity between *Anopheles* from the two West African sites, Burkina Faso and Guinea, is not different. This result for the prokaryotic microbiome is in contrast to the eukaryotic microbiome, where significant richness differences were not observed among countries sampled. Interestingly, despite geographic proximity between Burkina Faso and Guinea pools, both share more prokaryotic OTUs with larvae sampled from Kenyan pools than they do with one another which could be explained by the high species richness profiles in Kenyan sample (see Figure 4D).

Despite the fact that country of origin was the most significant covariate explaining microbiome composition for both the prokaryotic and eukaryotic microbiome, we also examined the influence of mosquito species after blocking for country. Sample sizes allowed for a comparison of diversity across *A. arabiensis, A. coluzzii*, and *A. gambiae* and showed that there was a significant difference in observed diversity for the eukaryotic microbiome, but no significant differences due to mosquito species for Shannon diversity of the eukaryotic microbiome nor for either observed or Shannon diversity of the prokaryotic microbiome (Supplementary Figure 5). Mosquito species is confounded with larval site ecology and dissection of their independent effects will require further work.

### Ecological Fluctuation of Eukaryotic OTU Abundance

**FIGURE 5:**
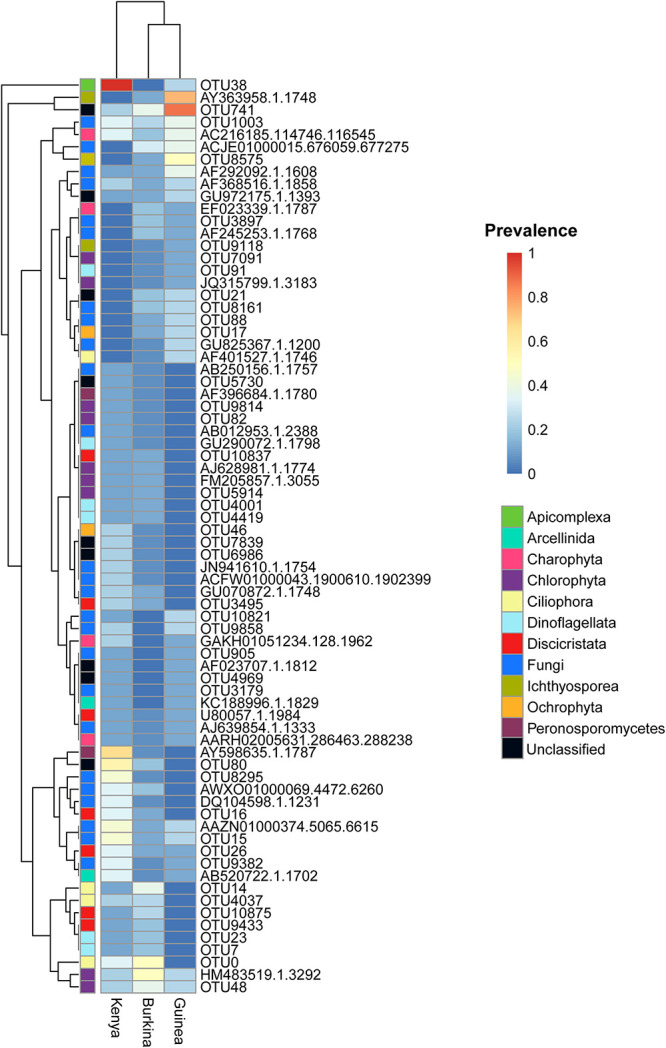
Eukaryotic OTUs detected in mosquito larvae display significant heterogeneity in abundance within and across countries. The heatmap includes the 74 eukaryotic OTUs detected in mosquito larvae collected in at least 2 countries. OTU prevalence is computed as the number of DNA pools with non-zero OTU abundance divided by the number of DNA pools from each country [*n* = 15 (Burkina), 8 (Guinea), and 9 (Kenya)]. The left most column is colored to indicate taxonomic ranks in the SILVA119 reference taxonomy. Black phylogenetic ranks correspond to unclassified OTUs by both sequence similarity and phylogenetic placement.

We analyzed the patterns of eukaryotic OTU prevalence among sample DNA pools grouped by country of origin (Figure 5). In particular, we wished to identify eukaryotic microbial taxa that could be consistent with an epidemic mode of spread and therefore suggestive of a potentially pathogenic microbe for mosquitoes. We filtered for taxa that fulfill two main criteria: (i) ecologically widespread, indicating taxa that may have had a large generalized impact upon mosquitoes and their ancestors, and (ii) fluctuating prevalence across individuals and geographic sites. The current data cannot exclude other possible explanations for this pattern, for example stochasticity or environmental heterogeneity, but it serves to prioritize candidates for follow-up studies. We also reasoned that ecologically widespread presence of an OTU could be a marker of an efficient colonizer, which could be easier to adapt to culture in the laboratory for biological studies of mosquito immunity and potential development as a biological control agent.

The eukaryotic OTUs display multiple patterns of ecological prevalence. Interestingly, a group of eukaryotic OTUs (Figure 5, displayed at the top of the prevalence heatmap) are highly prevalent in *Anopheles* larvae from Kenya and Guinea, and display fluctuating patterns of ecological prevalence. This group includes in the phylum Apicomplexa, OTU38_Ophryocystis; in the class Ichthyosporea, OTU_AY363958.1.1748; and the unclassified eukaryotic taxon, OTU741.

Each of these three fluctuating OTUs display prevalence at or near 1.0 in collections from at least one country, while at the same time being present across at least two of the three countries (OTU_AY363958.1.1748, DNA pools positive Burkina Faso 2/15, Guinea 6/8, Kenya 0/9; OTU741, DNA pools positive Burkina Faso 6/15, Guinea 7/8, Kenya 2/9; OTU38_Ophryocystis, DNA pools positive Burkina Faso 0/15, Guinea 2/8, Kenya 9/9). The OTU38_Ophryocystis is present in both East and West Africa, suggesting that its absence in the Burkina Faso sequences could be due to undersampling, while OTU_AY363958.1.1748 was only seen in the West African samples, which could be due to undersampling, or could suggest that its geographic range may not include East Africa.

The apicomplexan OTU38_Ophryocystis was described above. The OTU_AY363958.1.1748 belongs to the little-known opisthokont protist clade of Ichthyosporea, which is considered to be near the animal-fungal divergence, and which have been observed as parasites of fish and amphibians but also have relatives that are obligate arthropod gut endosymbionts (Marshall and Berbee, 2010; Reynolds et al., 2017; Borteiro et al., 2018). Finally, the unclassified OTU741 displays an almost complete match to the 18S rRNA gene amplicon sequence of an unidentified eukaryote generated from a soil environmental metagenomic survey [Blast score 193, percent nucleotide identity 99.07%, *e*-value 2e-45, 1701 bp, NCBI nucleotide accession number GenBank: MK945962.1 (Starr et al., 2019)]. Taxonomic placement and identification of OTU741 will require further analysis, as taxonomic databases for analysis of eukaryotic microbes are far less mature than those for prokaryotes.

### Effects of Laboratory Colonization and Adaptation on *Anopheles* Microbiomes

**FIGURE 6:**
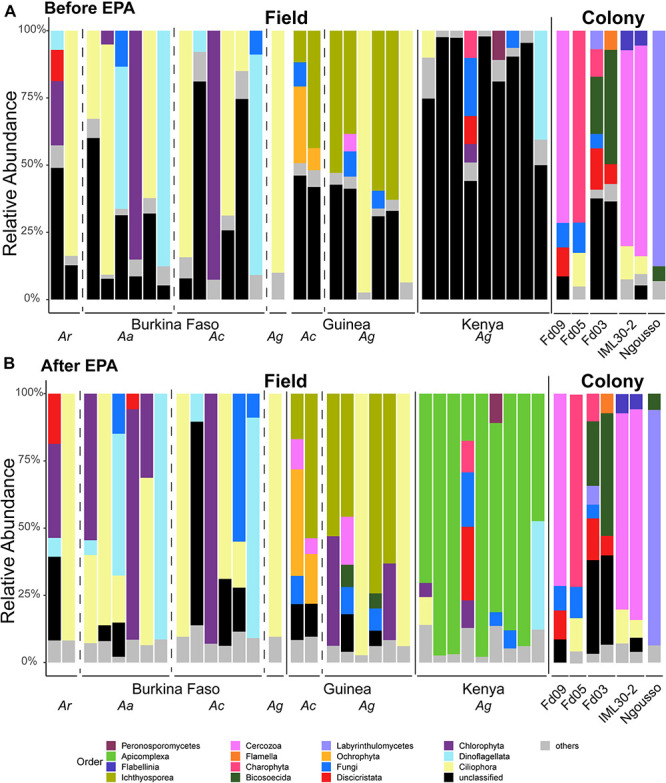
Taxonomic assignments of eukaryotic microbes are improved by employing sequence similarity and phylogenetic placement. Taxonomic Profiles of the micro eukaryotic members of the microbiome before **(A)** and after **(B)** application of the Evolutionary Placement Algorithm (EPA). OTUs are called at the Order level. All OTUs present at less than 5% frequency are grouped together and classified as others. Sequences for which there was not a Silva database match are termed unclassified. Each bar represents the micro eukaryotic taxonomic composition of one pool of larval samples from a given country or laboratory colony. Dashed vertical lines separate samples of different mosquito species (*Ar*, *Anopheles rufipes*; *Ac*, *Anopheles coluzzii*; *Ag*, *Anopheles gambiae*) and solid lines separate samples from different countries and = samples from the field from those from laboratory colonies. All colonies yielding eukaryotic microbes after rarefaction are *A. coluzzii*.

**FIGURE 7:**
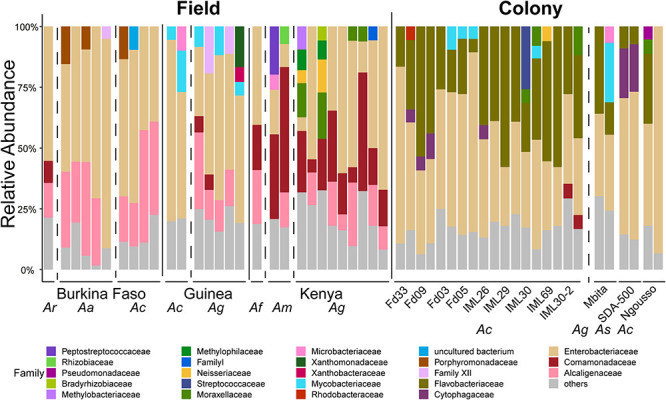
Taxonomic profiles of the prokaryotic microbiome in larvae sampled from the field and from laboratory colonies. OTUs are shown at the Family level. All OTUs present at less than 5% frequency are grouped together and classified as others. Each bar represents the prokaryotic taxonomic composition of one pool of larval samples from a given country or laboratory colony. All larval sample DNA pools contain only samples from a single mosquito species. Dashed vertical lines separate samples of different mosquito species (*Ar*, *Anopheles rufipes*; *Ac*, *Anopheles coluzzii*; *Ag*, *Anopheles gambiae; Af, Anopheles funestus; Am, Anopheles maculatus; As, Anopheles stephensi*) and solid lines separate samples from different countries and samples from the field from those from laboratory colonies.

We analyzed eukaryotic and prokaryotic microbial composition from larvae of insectary-maintained laboratory colonies and compared these to the composition of the field-caught larval samples (Figures 6, 7, respectively, and Supplementary Table 1). We first compare eukaryotic microbial profiles harbored by laboratory colonies to those of the field samples, and next compare microbial overlap between colonies and field samples, and finally compare microbial profiles among the laboratory colonies. Subsequently, the prokaryotic profiles are similarly analyzed.

**FIGURE 8:**
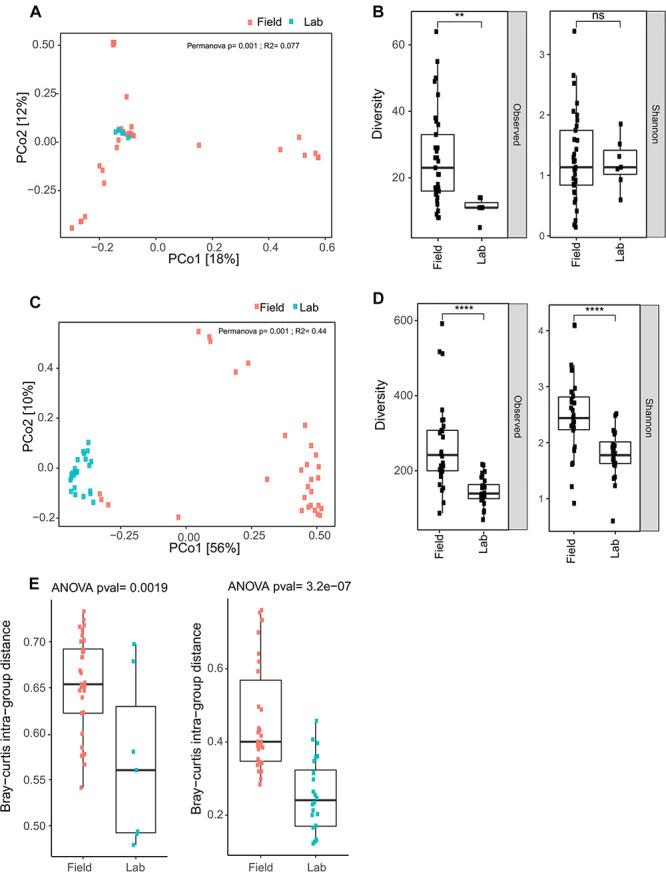
Field collected samples display greater eukaryotic and prokaryotic microbial diversity than laboratory colony samples. **(A)** Bray Curtis Principal Coordinate Analysis (PCoA) for eukaryotic OTU data reveal clustering of field and laboratory colony samples. The cluster of eukaryotic microbes in the colony samples is nested within the distribution of data points for the field samples. **(B)** Observed diversity of eukaryotic taxa is significantly greater in field collected samples as compared to laboratory colony samples, but there is no significant difference in Shannon diversity (ns, non-significant; *p* > 0.05; **, *p* ≤ 0.01). Upper and lower bounds of the box indicate the third and first quartiles, respectively, and the line within the box indicates the median. Whiskers extends to the largest values no further than 1.5 time the inter-quartile range from the corresponding upper and lower bounds of the box. All individual data points are shown as individual dots. **(C)** Bray Curtis PCoA for prokaryotic microbiome data displays distinct clustering of field and laboratory samples. Inter-sample diversity is less among colony samples as compared to field collected samples, as indicated by the tighter clustering of individual data points for the colony samples. **(D)** Both observed and Shannon diversity measures are significantly greater in field collected samples as compared to laboratory colony samples (****, *p* ≤ 0.0001). **(E)** Bray-Curtis intra-group distance, a measure of within group diversity is significantly greater for field samples as compared to laboratory colony samples for the eukaryotic microbial diversity (left panel) and for the prokaryotic microbial diversity (right panel), indicating greater difference among field samples than larval samples collected from laboratory colonies.

First, the composition of the eukaryotic microbes found in laboratory colonies is nested within the overall distribution of the field samples and do not form a distinct cluster, when analyzed qualitatively by Bray Curtis Principal Coordinate Analysis (PCoA, Figure 8A). Examination of the PCoA indicates that the field samples display greater variation across samples than their laboratory colony counterparts, which suggests less overlapping OTUS in field samples (Figure 8B, left panel). Interestingly, the Shannon diversity measure was not significantly different between field and colony samples (Figure 8B), indicating that a similarly low number of eukaryotic taxa predominate per sample in both field and colony contexts, regardless that the predominant taxa themselves are not necessarily the same between samples. This is also consistent with the impression taken from the taxonomic histograms (Figures 6A,B). The Shannon diversity result may suggest an inherent biological property of the eukaryotic microbiome, if these observations mean that only a limited number of eukaryotic microbial taxa can coexist within the ecological niche of a given *Anopheles* larvae. Further studies will be required to elaborate on these ecological and demographic properties of the eukaryotic microbiome.

Second, the overlap of eukaryotic microbial taxa is comprised of ten OTUs present in both field and laboratory colony samples at an abundance of ≥5 following rarefaction (Table 1). Four of the OTUs shared among field and colony samples are fungi. Of these, at least *Aspergillus* is probably present because the spores are ubiquitous aerosol environmental microbes. The other fungi present in both laboratory colonies and field samples are *Pleosporales*, *Wallemia*, and *Malassezia*. The *Pleospora* are a genus of ascomycete fungi. Both *Malassezia* and *Aspergillus* have been previously reported as members of the eukaryotic microbiome in *Aedes* larvae (Shelomi, 2019). The *Anopheles* laboratory colonies harbor an additional 16 OTUs present at an abundance ≥5 following rarefaction that were not detected in field samples in the current study (Table 1). These include two OTUs of the genus *Vannella*, which is an ameba found in soil and freshwater environmental samples (Smirnov et al., 2007). Five other taxa unique to the laboratory colonies belong to the clade termed “Stramenophiles, Alveolates and Rhizaria” (SAR), and include two members of the *Ciliophora* and two *Cercozoa*. These 16 colony-specific OTUs were detected in at least one laboratory colony and were not detected in any field samples, thus representing either rare natural taxa that expanded during colonization, or else taxa acquired during colonization.

**TABLE 1:** Eukaryotic OTUs in *Anopheles* laboratory colonies.

**OTU name**	**Taxonomic assignment**							**^#^col**	**Field samples**
AY183888.1.1959	Eukaryota	Amoebozoa	Discosea	Flabellinia	Vannellida	Vannella	NA	1	No
OTU9921	Eukaryota	Amoebozoa	Discosea	Flabellinia	Vannellida	Vannella	NA	1	No
EU186022.1.1834	Eukaryota	Amoebozoa	Gracilipodida	Flamella	Flamella arnhemensis	Flamella arnhemensis	Flamella arnhemensis	1	No
ACUP0100749 8.9215.11012	Eukaryota	Archaeplastida	Chloroplastida	Charophyta	NA	NA	NA	1	No
AGNK010040 45.34055.35850	Eukaryota	Archaeplastida	Chloroplastida	Charophyta	Magnoliophyta	Liliopsida	NA	1	No
U80057.1.1984	Eukaryota	Excavata	Discoba	Discicristata	Tetramitia	Naegleria	NA	1	Yes; BF
AY753597.1.20 86	Eukaryota	Excavata	Discoba	Discicristata	Neobodonida	Rhynchomonas	Rhynchomonas nasuta	2	No
OTU189	Eukaryota	Excavata	Discoba	Discicristata	Tetramitia	NA	NA	1	No
DQ104591.1.1252	Eukaryota	Opisthokonta	Holozoa	NA	NA	NA	NA	1	Yes; K
ACJE01000015. 676059.677275	Eukaryota	Opisthokonta	Nucletmycea	Fungi	Trichocomaceae	Aspergillus	NA	1	Yes; BF, G
OTU1003	Eukaryota	Opisthokonta	Nucletmycea	Fungi	Dothideomycetes	Pleosporales	NA	1	Yes; BF, G, K
OTU3897	Eukaryota	Opisthokonta	Nucletmycea	Fungi	Incertae Sedis	Wallemia	NA	1	Yes; BF
OTU8161	Eukaryota	Opisthokonta	Nucletmycea	Fungi	Incertae Sedis	Malassezia	Uncultured fungus	2	Yes; G
OTU9	Eukaryota	Opisthokonta	Nucletmycea	Fungi	Eurotiomycetes	NA	NA	2	No
OTU2074	Eukaryota	SAR	Alveolata	Ciliophora	Cyrtophoria	NA	NA	1	No
OTU3066	Eukaryota	SAR	Alveolata	Ciliophora	Colpodida	NA	NA	1	No
AJ514867.1.1767	Eukaryota	SAR	Rhizaria	Cercozoa	Rhizaspididae	Rhogostoma	NA	2	Yes; G
OTU1001	Eukaryota	SAR	Rhizaria	Cercozoa	Thecofilosea	uncultured	NA	1	No
OTU8372	Eukaryota	SAR	Rhizaria	Cercozoa	Rhizaspididae	Rhogostoma	NA	1	No
OTU2896	Eukaryota	SAR	Stramenopiles	Bicosoecida	NA	NA	NA	1	Yes; G
OTU69	Eukaryota	SAR	Stramenopiles	Bicosoecida	CH1-2B-3	Uncultured stramenopile	Uncultured stramenopile	1	Yes; G
OTU94	Eukaryota	SAR	Stramenopiles	Bicosoecida	Siluaniidae	Paramonas globosa	Paramonas globosa	1	No
OTU741	Unassigned	NA	NA	NA	NA	NA	NA	1	Yes; G
OTU18	Unassigned	NA	NA	NA	NA	NA	NA	3	No
OTU2085	Unassigned	NA	NA	NA	NA	NA	NA	1	No
OTU5832	Unassigned	NA	NA	NA	NA	NA	NA	1	No

*OTUs with abundance ≥5 following rarefaction.*

*^#^col, number of laboratory colonies with the OTU; field samples, present in samples from BF, Burkina Faso, K, Kenya, G, Guinea.*

**FIGURE 9:**
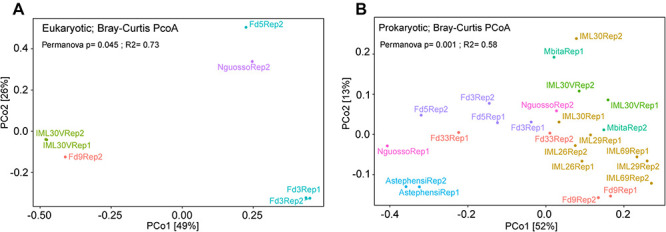
Principal Coordinate Analysis (PCoA) for microbial diversity in laboratory colonies of *Anopheles*. Co-housed *Anopheles* colonies maintain diversity for their **(A)** eukaryotic and **(B)** prokaryotic microbiomes. PCoA for the eukaryotic microbiome of seven colony samples with greater than 900 sequence reads following rarefaction. **(B)** PCoA for the prokaryotic microbiome of laboratory colonies. All colonies were sampled for both eukaryotic and prokaryotic microbiomes, but only those samples yielding adequate sequence information were analyzed (10,000 sequences per sample for 16S rRNA gene data and 900 sequences per sample for 18S rRNA gene data). Both plots highlight the similarity eukaryotic and prokaryotic microbial profiles across experimental replicates (all data points are labeled either Rep1 or Rep2). The variable tested in the Permanova was the colony of origin of lab pools.

Finally, comparing eukaryotic microbial profiles among laboratory colonies interestingly reveals that the eukaryotic microbiomes of these mosquito colonies remain distinct (Figure 9 and Table 1), despite the fact that the colonies are co-housed in the same insectary facility, and are exposed to the same water, food and other environmental variables. For example, colonies Fd09 and IML30-2 both harbored members of Cercozoa, a diverse group of heterotrophic protozoa that live in soil and freshwater, include pathogens of agricultural plants and aqua-cultured mollusks, and are also a component of the *Arabidopsis thaliana* eukaryotic microbiome (Braithwaite et al., 2018; Sapp et al., 2018; Irwin et al., 2019; Kang et al., 2019). The two replicate samples from colony Fd03 harbored high proportions of Bicosoecida, an order of unicellular flagellates including notable extremophile members adapted to low oxygen or high salt conditions (Yubuki et al., 2010; Harding and Simpson, 2018). The colony Fd05 predominantly harbored members of *Charophyta*, a group of freshwater green algae, including members with antioxidant activities (Kumar et al., 2015). The widely used Ngousso colony predominately harbored members of Labyrinthulomycetes, a group of protists that acquire resources by means of ectoplasmic slime nets, and include important pathogens of at least aqua-cultured mollusks (Rubin et al., 2014; Iwata and Honda, 2018).

Turning to the *Anopheles* prokaryotic microbial profiles, PCoA reveals that, differently from the eukaryotic microbiome, the prokaryotic microbial composition of the colony samples forms a largely distinct cluster from the field samples (Figure 8C). Observed OTU diversity is significantly higher in field as compared to colony samples, similar to the eukaryotic microbes. The observed number of prokaryotic OTUs for field samples averaged 264.31 across 29 samples (range: 81–601). For the laboratory colonies, observed prokaryotic OTUs average 148 across 24 samples (range: 69–219). Prokaryotic Shannon diversity is significantly higher in field samples as compared to colony samples (Figure 8D). The greater observed prokaryotic diversity in *Anopheles* field samples is also consistently detected by other indices of alpha diversity, including Chao1 (355 field to 227 laboratory colony), and Ace (367 field to 232 laboratory). These different alpha diversity metrics analyze evenness (Shannon) as well as species richness (Chao1, Ace). Notably, however, the laboratory colonies harbor similar proportions of rare prokaryotic OTUs (grouped together as “others,” gray bar in Figure 7) as the wild-caught field sample (Supplementary Table 6). These diversity results suggest that the prokaryotic microbiome may not display the same biological limit in the number of coexisting taxa per sample, as suggested above by the similarity of eukaryotic Shannon diversity between field and colony samples, and could indicate important differences in the biology of the eukaryotic and prokaryotic fractions of the *Anopheles* microbiome. Further work should focus on elucidating the meaning of these differences.

In comparing prokaryotic profiles between field and colony samples, members of the *Enterobacteriaceae* are predominant at the family level in both sample types (Figure 7). The *Enterobacteriaceae* are gram-negative bacteria that include *Salmonella*, *Escherichia*, *Klebsiella* and *Shigella*. The betaproteobacteria families of *Comamonadaceae* and *Alcaligenaceae* are present at >5% in many field samples but are largely absent from laboratory samples. *Flavobacteriaceae*, a family of Bacteroidetes are present above 5% in only laboratory colony samples.

To quantify differences in microbial composition among field and laboratory colony samples, we measured the Bray-Curtis intra-group distance. For both the eukaryotic and prokaryotic microbes, this measure is significantly higher in field samples as compared to laboratory colony samples (Figure 8E). Thus, the compositional profiles of both eukaryotic and prokaryotic microbiomes are significantly different between field and laboratory colony samples, with field samples exhibiting greater variability. These results indicate that *Anopheles* larvae sampled in the field display greater inter-sample difference than do larval samples collected from laboratory colonies.

## Discussion

Comprehensive mosquito sampling and characterization of the eukaryotic and prokaryotic microbiomes generated a number of new findings. First, geography is the strongest correlate of microbiome composition at both the eukaryotic and prokaryotic levels. Country of sample collection has greater explanatory power for microbial diversity than mosquito species, larval site type or larval site ecology. Second, there are significant differences in both eukaryotic and prokaryotic microbiome diversity both among and between field and laboratory colony samples with field samples showing both greater overall diversity and greater across sample variability. Despite these field and laboratory colony differences, there are shared OTUs present in both field and laboratory colony samples. Wild OTUs that are found in laboratory colonies may provide a convenient opportunity for mechanistic studies of microbiome interactions with the host. The comprehensive description of microbiome composition presented may offer candidates for new malaria control tools, including classic biological control agents.

The findings of both the eukaryotic and prokaryotic microbiomes confirm trends that have been reported previously only for the mosquito prokaryotic microbiome. Environment appears to exert a larger influence on shaping the mosquito microbiome than does the genetics of species differences (Yun et al., 2014; Rothschild et al., 2018). Nevertheless, genetically distinct laboratory colonies maintained in a shared controlled environment may display stable microbiome differences based on genetics or on vertical transmission via the larval rearing environment. Multiple pieces of evidence support the finding that mosquitoes acquire their microbiome each generation from their environment. This evidence includes extensive overlap of microbial composition between mosquito larval and their aquatic habitat (Boissiere et al., 2012; Coon et al., 2014; Gimonneau et al., 2014; Buck et al., 2016), a lack of microbes in the gut of newly hatched larvae (Coon et al., 2014) and extensive variability in mosquito midgut communities which would be unlikely if microbiomes were acquired from parents (Boissiere et al., 2012; Gimonneau et al., 2014; Buck et al., 2016). Thus, although environment may play a predominant role in determining the array of ambient taxa that a newly hatched larva will encounter, our current findings also suggest that host genetics may play an important part in shaping the precise community of taxa that persist within the host. The possibility and basis of stable differences for microbial profiles among co-housed mosquito colonies deserves attention in further work.

### Deriving Value From the Eukaryotic Microbiome

With comprehensive characterization of eukaryotic microbiome members in both field samples and laboratory colony samples, efforts can shift to more mechanistic understandings of the role of these microbiome members and how they are balanced with prokaryotic and viral members of the microbiome. Future vector control efforts could potentially use apicomplexan members of the mosquito eukaryotic microbiome that may compete with *Plasmodium* superinfection, or as biological control agents for population control. The apicomplexan OTU38_Ophryocystis is widespread in Kenyan larval samples, but was not detected in laboratory colony samples. There are other eukaryotic taxa associated with laboratory colonies that could be exploited in mechanistic studies, including ten eukaryotic OTUs that are also present in field samples.

Little work has been published on the eukaryotic microbiome of mosquitoes. This is largely due to the greater technical challenge in separating eukaryotic microbial 18S rRNA sequences from the mosquito host or vertebrate bloodmeal (Belda et al., 2017). There are reports of fungal isolation from *Anopheles* larvae and adults using standard microbiological techniques (Ricci et al., 2011a, b; Bozic et al., 2017) as well as published results suggesting fungus can have negative (Bargielowski and Koella, 2009) and positive effect on *Plasmodium* infectivity (Anglero-Rodriguez et al., 2016). The current work examines the composition of eukaryotic microbiome taxa through similarity with available 18S rRNA sequence databases, which are less mature as compared to those for prokaryotes. To circumvent this limitation, we generated taxonomic assignments based on sequence similarity and phylogenetic placement. Application of this analytical approach allowed identification of a large fraction of novel eukaryotic microbiome OTUs found in *Anopheles*. Moreover, these results highlight that almost all of the unassigned OTUs detected in field samples from Kenya belong to the phylum Apicomplexa as OTU38_Ophryocystis.

New vector-based tools are needed to bolster efforts toward malaria elimination and eradication. The *Anopheles* prokaryotic microbiome has been reasonably well characterized, and has yielded candidates for malaria vector or transmission control. Both natural and genetically modified microbes as well as Wolbachia have been shown to block parasite transmission (Gao H. et al., 2020). In comparison, studies of the eukaryotic microbiome are in their infancy and more studies are necessary to understand the biology of the eukaryotic microbiome. More data on the eukaryotic microbiome as well as the mosquito virome will inform the tripartite interactions between prokaryotes, eukaryotes and viruses that together shape the ecological community of commensal, symbiotic and pathogenic microorganisms affecting mosquito physiology and vectorial capacity.

## Data Availability Statement

All sequence files are available from the EBI European Nucleotide Archive database (http://www.ebi.ac.uk/ena/) under ENA study accession number PRJEB40885. Assembled eukaryotic OTU 18S rRNA amplicon sequences are available in this article, Supplementary File 1, and assembled prokaryotic OTU 16S rRNA OTU sequences, Supplementary File 2.

## Ethics Statement

The animal study was reviewed and approved by the research animal ethics committee of the Institut Pasteur, ‘C2EA-89 CETEA Institut Pasteur’ as protocol number B75-15-31. The Institut Pasteur ethics committee is authorized by the French law N° 2001-486, which is aligned with Directive 2010/63/EU of the European Commission on the protection of animals used for scientific purposes.

## Author Contributions

EC, KV, and MR: designed the research and wrote the manuscript. EC, BC, TB, KE, MC, SZ, MB, AG, WG, AB, ST, N’FS, and MR: performed the research. EC and MR: analyzed the data. All authors contributed to the article and approved the submitted version.

## Conflict of Interest

The authors declare that the research was conducted in the absence of any commercial or financial relationships that could be construed as a potential conflict of interest.

## Supplementary Material

Supplementary Figure 1 Quality profile plots of raw sequence files and assembled amplicons of pools included in the study from FASTQC quality checking. **(A)** Quality profile of forward (R1) and reverse (R2) Illumina 16s MiSeq reads before the Pandaseq assembly step. Each line depicts the mean quality score of read positions with 10 and 90% quantiles depicted as whiskers. Background color signifies FASQC standards for good (green), reasonable (yellow) and poor (red/pink) quality. **(B)** Quality profile of the 16S amplicons assembled by Pandaseq. **(C)** Quality profile of forward (R1) and reverse (R2) Illumina 18s MiSeq reads before the Pandaseq assembly step. **(D)** Quality profile of the 18S amplicons assembled by Pandaseq.

Supplementary Figure 2 Rarefaction analyses of field and laboratory colony DNA pools. Rarefaction plots of 16S rRNA gene **(A)** and 18S rRNA gene **(B)** datasets based on 4 alpha-diversity indexes (Observed Species, ACE, Chao1, Shannon). Curves are colored by the source of the sample (laboratory colony samples, orange; field samples, blue). Vertical dashed lines correspond to the threshold of rarefaction fixed in the analyses to correct for differences in sequencing depth (10,000 sequences per sample for 16S rRNA gene data and 900 sequences per sample for 18S rRNA gene data).

Supplementary Figure 3 Evaluation of PNA blocking efficiency on 18S rRNA gene data: Bar plots representing the relative abundances of Insecta (orange) and non-Insecta (blue) sequences in 18S datasets of mosquito DNA pools stratified by source (laboratory colony, field). Those samples with Xs above the bars had inadequate numbers of 18S reads for analysis and were not analyzed further. The failure of laboratory colony samples to provide ample non-mosquito 18S sequence reads was not due to low sequence depth as colony samples were sequenced to equal or greater depths than field samples.

Supplementary Figure 4 Water blanks show no significant contamination affecting either eukaryotic or prokaryotic microbiome results. **(A)** The 18S read abundance of non-insecta reads from all samples for all taxa detected in water blank samples. **(B)** The 18S read abundance for only those samples that passed rarefraction and were analyzed in the paper. In neither **A** nor **B** are there any 18S eukaryotic microbial reads in experimental samples that were derived from water controls. **(C)** 16S read abundance for all reads with reads separate by those also detected in water control samples and those absent from water control samples. **(D)** As in C, but only for those samples that passed rarefraction and were analyzed in the paper.

Supplementary Figure 5 Diversity profiles of field pools across different mosquito species. **(A)** 18s OTU richness distributions across mosquito species. **(B)** 18s Shannon diversity distributions across mosquito species. **(C)** 16s OTU richness distributions across mosquito species. **(D)** 16s Shannon diversity distributions across mosquito species. Statistical comparisons were carried out between A. coluzzii, A. arabiensis and A. gambiae (those species with sample sizes greater than three per group) by means of a Kruskal–Wallis non-parametric test blocked for country of origin using the independence_test function of the coin R package.

Supplementary Table 1 Description of pooled larval samples used for 16S and 18S amplicon sequencing.

Supplementary Table 2 Eukaryotic OTUs before application of the EPA.

Supplementary Table 3 Eukaryotic OTUs after application of the EPA.

Supplementary Table 4 Prokaryotic OTUs.

Supplementary Table 5 Diversity measures of eukaryotic OTUs.

Supplementary Table 6 Diversity measures of prokaryotic OTUs.

Supplementary File 1 Fasta file of eukaryotic OTU sequences.

Supplementary File 2 Fasta file of prokaryotic OTU sequences.
